# The incidence of malignant neoplasms in Jamaica.

**DOI:** 10.1038/bjc.1965.82

**Published:** 1965-12

**Authors:** G. Bras, D. C. Watler, A. Ashmeade-Dyer


					
681

THE INCIDENCE OF MALIGNANT NEOPLASMS IN JAMAICA

G. BRAS, D. C. WATLER AND A. ASHMEADE-DYER

From the Jamaica Cancer Registry, Department of Pathology, University of the West Indies,

Jamaica

Received for publication June 24, 1965

To determine cancer incidences accurately it is necessary to have adequate
diagnostic facilities available to the population at risk, as well as demographic
data on that population. The Jamaica Cancer Registry therefore restricts its
activity to that region of Jamaica where these desiderata are fulfilled. Figures
accumulated over a six-year period-1958-1963 inclusive-are now presented.

FiG. 1.-The island of Jamaica; elevations above sea level are shown.

Geographical data.-Fig. 1 shows an outline of Jamaica giving elevations
above sea level, Fig. 2 gives details on the parishes served by the Cancer Registry;
these data include rock types, rainfall, background gamma radiation in micro
roentgen per hour. Some climatological data are grouped in Table I.

Kingston is in the rain shadow of the Blue Mountains (peak 7430 ft high),
where moist North East trade winds lose most of their water. Consequently

TABLE I.-Climatological Data for Kingston*

Temperature in degrees F.
Mean

Hottest monthly average
Coolest   ,.      ..
Annual range
Highest ever
Coolest ever

Humidity                  Sunshine

79  . Mean   .    .   . 80%    . Jan.-March 70-72% (Max.)
81  . Highest month (Oct.) 84%  . Sept.-Nov. 50-57% (Min.)
76  . Lowest  ,, (July) 76%      .
5
97
50

* Average of readings over 33 years, 4 times daily, at elevation of 100 ft.

(6T. BRAS, D. C' WATLER AND A. ASHMEADE-DYER

I:'    ,._._ . >7645' PARISHES OF KINGSTON

AND ST ANDREW

Io 0,0 -.

HILLY''

J"SURFACE '
80' DRAINAGE X

-5....,GRANITE~..N

HILLY.

NO SURFACE DRAINAGE -                ou,

.LIMESTONE

KJ1,               \\ NON CARBONATE ROCKS      X

FLAT

\..*PERIODIC SURFAQEF LQRAINAGE  \               .

~**ALLUVIAL GRAVEL.*.

FI(G. 2.- Some dlata on the parishes of Kingston and St. Andrews including rock typles, rainfall inl iocht's

1)e anlnum, and (circ?led) backgrouncl gammua radiation in micro roentgen pd'l hour.

whlereas the rainfall in the Blue Mountains is 200} in. per annum it is less thaii 40t inl.
per annum in the city of Kingston.

Two rainy periods occur respectively in Mlay and October and most rainl fall;s
in torrential downpours. D)roughts causing serious water shortages occurred
frequently before modern hydrological methods came into use approximately
:30 years ago.

D)emographical data.-Data provided by Roberts (1957) show that the popula-
tion of Jamaica has since 1844 retained a greater degree of homogeneity than most
of the other populations of the W'est Indies; there was only a small amount of
muigration after cessation of the slave trade. Ever since 1881, when full racial
breakdowns first became available, 9600 in the population returns were made up
by those groups designated as black (Negroes) and as coloured (mainly the product
of Negro and E,uropean inter mixture) ; these groups have conlsistently made up

I

6 8

MALIGNANT NEOPLASMS IN JAMAICA                              683

respectively 7800, and 18%    of the total population, which betweeni 1844 and 1960
rose from  377,433 to 1*6 million.

'r'AlILE II.-Poptlation of Kingyston and St. Andrewv ('ensus, 196(0

Age group)        Male          Female

0-4            32,373         32,078
5 -9           23,197         23,933
10-14           17,032         19,012
15-19           17,108         22,816
20-- 24         17,865         23,712
25-21)          16,151         21,654
30--34          13,145         17,155
35-39           11,805.        15,743
40-44           10,343         12,222
45-49)           9,342         11,085
50 54            7,387          9,084
.)t-55 51)       5,091          6,565
60 64            3,686          5,012
65-69            2),051         3,277
70-74            1.451          2,538
75-79              904          1,775
80 +              837           1,987

Total - 1891,768 Total - 229,648

Of special interest for our Cancer Registry is an analysis of racial elements in
the Kingston and St. Andrew area. Roberts' figures show that Whites are the
most urban with 60% of their numbers in Kingston and St. Andrew, followed
by Chinese (50?/), Coloured (30%0), East Indian (20%/), Negroes (16%).             This
means that in the Kingston and St. Andrew population-having approximately -

of the total island's population-Negroes make up 65% and Coloured 30%,1 of the

aage

80+
75-

70-74
65-69
_60-6

55-59

Males                       5-54                                Females

45-49
40-44
35-3
30-34
25-29
20-24
15-19
10-4

0-4

35    30

25   20   15    10    5      0    0     5     10    15   20   25    30   35
Fi'c,. 3. -The .Jaiiaiean population (in thousainds) durinig the 1960 census.

G. BRAS, D. C. WATLER AND A. ASHMEADE-DYER

-  o O

sz   0 |   %  Qi  e >bD1: X

s- >  o-Z ,0    l

O0 C

o   b

.2  . It  0 (Z M t- 00 t-  N  -

C C o  o -o  t- O q ?.   m  00 C sQ o   C _

40     0  $  t  -<

CD ~ ~ ~ ~ ~ ~~~~~L C)00MC)

OZ 0

0      e

o                   M     W km C :

0D

Er; IZ-  =s  0

bo   ,~~ 0 0 I.0

0                     -

*4 0' C G  4?  Q M Oo

| > fl <, n @ O e C4 C l S] M _ IC _

H   o

';      b   biD?   -Ot : <- :t X Xt

0          C -   -  -  -C
HZ  0  L  ,  c

xo~~ ~~     CZ  to Q  00

.-C

tD~~ ~~~ O- o '- (m in .- oo 5 t NbXs   m o  O' an

O~~~~

0~~~~~~~~~~~~~~~~~~~~0

? 9   ? ? t-  *O
0  0 rt. -d  km N t- - N Lt C  -eto

_4   _    _4 _I

A0
E-4  s   z O z

0_  _0 _

684

MALIGNANT NEOPLASMS IN JAMAICA

population in this area. The remaining 500 is made up approximately as follows:
Chinese 122%, East Indians 0.4%, Europeans 0.3%, others 3-1%.

Occupational data.-" Unfortunately, in analysing occupational data, we enter
a domain in which census material proves often treacherous and unrewarding"
(Roberts, 1957, p. 85). Figures available show the following sub-division of the
working force: professional 500, domestic (personal) 14%, commercial 11%,
agricultural 44%, industrial 26%. Industrialization, however, is progressing
rapidly and the agricultural labour force decreasing concurrently.

Literacy proportions (percentage of population over 5 able to read and write)
is above 90% of the Kingston and St. Andrew area, but even in the most backward
parishes the figure is not below 60%.

Organization of the Registry and Results

The Registry is centred at the University-just outside Kingston. It records
all cases diagnosed in the Kingston and St. Andrew hospitals. This area is served
by several well equipped hospitals and the Registry staff collects data on cancer
from all hospitals, nursing homes and medical practitioners (Table III).

Returns from the Registrar General are compared with and provide a cross-
check with the figures so obtained.

Histological confirmation for the cancer diagnosis was obtained in 7900 of the
male cases and 85% of the female cases (Table IV). Where no histology was
available the diagnosis was only accepted on combined clinical and radiological
evidence.

The patient's permanent address was carefully checked and, for purposes of
the incidence study reported here, only residents of Kingston and St. Andrew
were included.

During 1958-1963 a total of 2898 malignant neoplasms were registered (Table
V); Table VI gives data for each year of operation.

Comparison with other countries

Tables VII and VIII compare some of our findings with data from Denmark
(Clemmesen and Nielsen, 1952) and from African Bantus (Higginson and Oettle,
1960).

The following seem worthy of further comment: Oesophageal carcinoma has a
high incidence, as in the Bantu. Primary carcinoma of the liver is much rarer
than in the Bantu, but more common than in Denmark. Carcinoma of stomach
is the single most frequent tumour in the Jamaican male; gastro-intestinal cancers
are more frequent than in the Bantu but below the Danish figures. Cancer of the
skin is remarkably common in Jamaica when compared with the Bantus. Repro-
ductive organs. Penis carcinoma is common in Jamaica as it is in the Bantu and
so is carcinoma of the cervix uteri; (circumcision is not common in Jamaican
males). Testis cancers are rare in Jamaicans and Bantus. Breast cancer is
common in Jamaica and Denmark, rarer in the Bantu.

SUMMARY

The Cancer Registry in Jamaica has accumulated complete data on a population
of 420,000 in whom, during 1958-1963 inclusive, a total of 2898 malignant
neoplasms were observed. Table V shows the distribution of these tumours by

685

G. BRAS, D. C. WATLER AND A. ASHMEADE-DYER

0

, o                       00       to o   :<n

s   -       :<oo_oooooo> o o   oXoo

o4) 4a0-0000 N 000 00

bO.f ?~ =  N X 0 X o o o 00 0  o o 0   0  o 0 o0 c o  N N 0 5

. . . . . . . . . . . . . . . . . . . . . . . . .

0 6

m.~ o s N I I  I  I -  I  I  I I I 1 1

4. 0X _

0  ~ ~ 0

6   4

z

0     I   Ii I

t

0o

0

0    I I  I I  II

m00  s 1 1 1 1 1 1 1 1 1 1 1 1 1

3C.)   1   ?   I  - I  I,

v   .

-0      = m X

=O 0] 10<

10) 10 0) 11 II =4 000

- N-    - (V. m   -      00 (

IX r I cM t 3wt c e

I        l-

I 0-o _ q - a- cc  -
I v,0t-,-      -

1-   1? I  10?<  I - 1I I I

I       I - 11 11 11

I  I  I  I  I  N-  I   I  - ! I 'O-  I  I

o0          0]-8? -      -            X             e  si om    0_

. . . . . . . . . . . . . . . . . . . . . . . . . . . . . . . . . . . .

0  .

bi                                                 -4 D

.SB.
X 3E _ fiS           8        0          0 s_*,

.   0 .  .   .   .   .   .   .   .   .   .   .   .   .   .   . Z

~~~  0  0~~~~~~~  ~~~  ~ ~ ~ ~   0~P

Z (

0 I

4-a I

. - O

--

-1        1

686

---

q4)
4.)

CO

I-)

"IC;N

CO
*  ;

:;
?4

0   -_   C1  C0  't  IC    0   N r      0o  -o   C]  C c

-It -t  IRt It   It  -t    do   4       10 to  10  IC  10  IC

-     -? -     -    -   -   -    -   -    -   -   -        -

I

I-* li? N =
1 4 N

MALIGNANT NEOPLASMS IN JAMAICA

000  0 1 o   C   o  C  I0 o   0I

o:obrsI X ooIG

. . . . . . . . . . . . .
I  I  Iev  IC ?  I  je 10

I 00 m b:-  1  0~~~~~~~ Ir

01 +4

I -I  I  I  1III 1 1

I  1   COI: -  I 1-'  1?

0

Io   I o

I:~ I o

1 K

I   --  I 'll"
I la I _

Ill, I I

*- b.

I I I  I X

. . . . . . .

II    1 1 I '

I  I  I X   I cc  r
I I I "I, II Is

II   I   I   I   I   l

II I - I Moo

Z?   ?   COCON  10?

I 00o??
I ? I? ,00-00010-

I- K I'?'? I K

CO?CO10
I  ?    0101  10C0  -

I I  10  j01"?  1010

_~ 0  _   0 0   o o 0   m   1 C '   -   1 0 0 0 0 0 0 0   *   0   r   01   0 1 0 1   s   _ - X   1   0   C tO   0   O   0 0 1 -

bo                   C    1                                                    -  -

0~~~~~~~~~~~~~~~~~~~

*    *   0   =   .....  >X?                                 -

S  wO  E  WYt  S  zO:  l  X  e ;  g  F}iS<c     X    'C  ck   ]vm>     a         X~~~~~~~~~~~~~~~~~~~~~~~~~~~~~~~~I  C

0                                .                          .                      0

>                  ce o :e   o-o-mc?

*                      -         .         -            0   0

(                 U)  ,                                                  1

~~~~~~~~~~~~~~~~~~~~~~~~~~~~~~~;4--                                      0.4~o. >

-   U )   0 0 0    ~~~ ~~~~~ ~~~~~~~~~~~~~~~   0   0   ~ ~ ~ ~ ~ ~ ~ ~ ~ ~ ~ ~ ~   -~~~~~0   - O-   0.   0

-   0 0~~~~~~~~~~~~~~~~                0 S~~~~~~~~~~~~~ o o   U)~~~~~~~~~~~~~t   0- 0   0 (   M (

~~~   0    0   1~~~~~~~~~~~~   0   0   -~~~~~~~~~~~~~   -~~~~~~   ~ ~ ~ ~   ~ ~ ~ ~   04  -  - 0   1 4 - 4  4 -   0-

687

0

0

I  I

I-

I I

I  I

r- -

L- L10,

I= m

,--

I I
1 I"

688

G. BRAS, D. C. WATLER AND A. ASHMEADE-DYER

0~~~~~~~~~~~c

D    .  0  0 t- 00 o  <  0 >  t  0    N _  00

-E ."  o               ---     ----  -  -

0 a

I I

m ~                               - -000x 4  ( ZC  C0mt

--4-1       -   -r  -  0   -  e  -4r4 I4 0
oo

o. bi  4

0  24 I   I I  Ij     jIN              i
0

0    ~

0 0~~~~~~~~~~~

H     0 ..                                o

IC  .~ ~ ~ ~ ~ ~ ~ ~ ~ ~ ~ ~ ~ ~ ~~

0 ~~~

0  cq  N 0   .     .  .

0                S

04 5
-      4

0 ~~~~04       5 4

z 9 o   i;           e              0  m  t_

0     O         -   01     0  - (

~~  kC~~  CO  N  ~~~  0  0  0  0  -  0~~~ 1  4  ( t   0

a  S   f  I- 1-1 -   -  -  - 1  1 01  01 1  1 1  1 1 1  0  0'  1 0 1 01  1 -X  011
Y~~~~~~~~~~~~~~~~~~~~~~~~~~~( -
I ~ ~ ~  ~   ~   ~   ~   4     4

-~~~~~~~~~~~~~~~~~~~~~~~~~~C       0
e ~~~~~~~~~~~~~~~~~~~o  O  X  :<+s_sn Q:

-4   -4  o.               04  N  N  aq  P o

MALIGNANT NEOPLASMS IN JAMAICA

co

- e 0 I  0I 11- 1   - 1C~ 1 1- -+" 111  11111

--
0

10

?  t-?  I I I --  1  1- 1 -

o

k"o InH~

km I
1 0 k   to

,   I  I  I - -I  I  I _ _  I

n  1--I  Q   I-  I - I I  I
1 0  0  _ 1 1 _ _ _   I  I

km _
km m

m 00

cq *  Xn  11

0 -

:4 10 111 11

0 i x  l  l 1 1  1   1 1 1 1  1  1

I I I I

I Is I I

I Cq  -4

I   I -        I

I 1 -4 -

- l  I   I   I

I    I   I   I

I -     I    I

lI I I
lI I I

I   I   I    I
I   I   I    I

H   [ 0 C O N

CO 104
I-
I  cq

- CO ~010 COO

I CO? CO101 K'"?I II'?

I I I I I I

?00101 10101 I K10'

I z0'?01- I 0101 j j

I CO?CO0100110?? CO

t-CO?401

I 10O1?0CO0110?4 J01??4

I I

10CO.?4

04,

t~        ~~                     . o o

f o

001~~~~~~~~~~~~~~~~~~~~~~~~~~~~0m0

0t   0  -?|#   01#   C OlX   's 4  10  0   -l   C O  0   01  C O  ' U  1 0  1 0  t   h '  CO

_   1  1 0  1 0  1 0  1 0_  1 0  1 0  1 0  1   1 0

04

0

bo

I-

bD

CA)

QC

0

0
'At

0q

m.

?

689

G. BRAS, D. C. WATLER AND A. ASHMEADE-DYER

00  0 0 t  Cq  C _  O0 0  0L 00 0 0  0 0 0 C  t: 0 0 O C  0 0  C: 0  CC. N.  ?1 C C.,  0
_ _         ~    l oN  '>l Cil  N  CiN  t -  (>1  t-C:4CD "C   -O
- ~ ~ ~ c  ooC] ;>                             - C  -

bO

oil    S  SSx>>xWut>>rWSSSSS

0

-4-

I  I~  I- 1I   I   I""1  I   I   I  I  I  I  I "l II   I I  I

111 Ic Io - I

I II~~~~~~I I a

II KI~~~~I~~CiI ~~ C=q

~~  Ci~~I0~~  I

a        IV

IIQ     I

II1 ~
I11I1 I

II1 II-

I~ K I I
I~ C I I

I~ NI t-IC

I o:I  K e
I eDI I K

I rI I I c

NII   I  K

1111 ll

111111 l
111111 l

0

0          0          C)
1x         M          0

a1)        0          0

Q            Q          Q

I KI?I KI

I I10?I?? I10??CCI

10    j     j            I

K K' Ci 110 Ci I'?'?'? I

I    C?10  j  j Ci  j

Ci   j 10     - j -?

I I?I?'I 11111 11I1 II
111111111 I I?'I liii INN

0  ?                0
?0

?

? 0 "e

00

S

*-      O     -    N1   "    O    -   ,I C1     m '   10 Cl      N -  0   =    0    -    0    -    Ci1

e               e.   e    t-   t    t-   t- tN- N       t-   t-   t   - N              0)   0    0 )

_    _-  -_    _4   _4   _0   _4   _4   -_   _.   _-4  14l                        4  I

690

a4
0
0
baD

?)

I

to
10

0
0

t-

0

ul

I

0

I

10

ull
t1
0
to

0
0

Ci
~t

I

to
m

C>

1~

CQ

_S
_O
8
sO

0
EP

Ei

H~

e

MALIGNANT NEOPLASMS IN JAMAICA  691

N  00 0  1i  i I i  I  f 't  -   -   t- C   4   C  C  co 0  I  o  0 oo  O

Ci   C    PC' 4 -P-j4 O Ci"  Cl -  "   C 0  = -i'-4

-o t~-

I  I  t  I  I  I I --  -  IfI  I  I   I  , I i o l I

I   l  i  I   "'1~ ' 1  --   Il t 1 t t ' I  I  I '1  1

I I  I -'  I  - 1 t ~_- I  I  I I  , I  I  I -  I '  I  I

:i     K' ~  ! t~'- , -KIi'I'  - t'!  I   K I  I~   '
-I  !  - i... III Ii I  I ! Z I  C? 1-

C4i

' K '-=  I     _iC  ' _4  I  I I 1  t  I I~ICl I~CC

' ~ ' t' l' ~ " ~-~ ' l'~ ~'~'i'~~~~~~~~~~~p  Cl'  I   Cl 9
_ 1--   ,  ' i-I-   M II I *   I I  I

- 'i I-i I- I  I..  I  _    I  I I ICC- ' I
~' 1''1-  ,-, ! t'  -t- I  1'~'" I't !'- -!x "-
__  ~I' ~ II _   1  "_  I  I  I  I  I !  tI~I'  1I

__ I+ iI - I-- II -  IfC'lCl !  I,tiIIIIIj?  I - ,,  I

I _t_I I _ I'  j 'I t II,t, I _ I  I  I  I I I  I 1t -' 1 1 1 _I

I .  . ,  I ~ , ,..,I....I..II  -  -4t>tF

~ ~

0 ~

_ ~ ~ _  I5 I -  __ I- III II- III- IIIIIIIII -  0  b

o ~      ,>      o

00

0 ~ ~ ~ ~ ~ 0   C

t.C,  +  <   0'D  b  iX  C5>  0  0  0  -  CD  CC P .  0  0

r....

>  0      0 0 0 0         0  0 00 0 4

?Ci             Cl s  3u X  :, ,=  o o  oCi o  o  o  o  C-

- ---  -4  t .  q  t  e.11  (C.1  tS  C9  't  Nt  N  _   N

9

G. BRAS, D. C. WATLER AND A. ASHMEADE-DYER

TABLE VI.-Total Number of New Cancer Cases Diagnosed for the

Period 1958-63 by Primary Site, Sex and Year

1958    1959   1960    1961   1962    1963   Total

. Lip  .

. Tongue

Salivary glands
Floor of mouth

Mouth unspecified
. Mesopharynx
. Nasopharynx

. Hyphopharynx.

Pharynx unspecifie(l
O Gesophagus
. Stomach

. Small intestine
. Colon

. Rectum

Liver (10)

Biliary passages.
Liver (20)
. Pancreas

. Peritoneum

M..     M

F.

M..           .

F.
M..     M

F .

M..           .

F.
M..     M

F.

M..    M

F.
M..     M

F .

M..     M

F.
M..     M

F  .

M..     M

F .

M..    M

F.
M..     M

F .

M..     M

F.
M..    M

F .

M..     M

F .

M..     M

F.
M..     M

F .

M..           .

F'.

M..           .

F.

Unspecified digestive organs  M.

F.
Nose, nasal cavities, middle  M.

ear, sinuses  .    .     . F.
Larynx     .    .    .     . M.

F.
Bronchus, lung (1 )  .     . M.

F.

Mediastinum     .    .     . M.

F.
Breast    .     .    .     . M.

F.
Cervic uteri    .    .     . M.

F.
Corpus uteri    .    .     . M.

F.
Chorio carcinoma     .     . M.

F.
Uterus unspecified   .     . M.

F.
Ovary, tube and broad         M.

ligament                    F.
Other unspecified female      M.

organs                      F.

1

0
4
1
0
1
0
1

2

5
2
0
1

0

1
1

11

7
21
18

1
0

5

10
4
7
6
0
6

5

3
0
5
3

3

3

1
14

1

39
85

8

4

0

14

2
3
2

1.

1.
1.
1

3
12

5
21
16

8
14
3

5.

4
2
7

3

3

1 4

2.

1.

53
83

3.
3.

1 2

4

1
3
3
3
3

1.

1.

12

3

18
10

29
12

5
13

6.

12

5.

3
3

4.

2
4

11

6.

58
97

7.
4.

12

1

1
3

1

2
2

5

1

1

17

5
35

9

9
12
5
11
4
1
1

1

1
6
I
1
3

9
6
1
2

54
82

6
5

9

7

1.    -   .   3

_     1 .    5

2  .   4   .  20

2  .       .  10

2 .    7
2  .   3   .  11

.   1 .    4

* 1 .    4

4      9)
I            I)  I

1

1 .
14.

6
26
21

11
20

3
8
4

3
3

5.

2.
2.
6.

19

4.
1.

.55
92
10

1.

9.
6.

2  .   1 I

9
3
I      87

1  .    1
1   .  87

6_ . 39

28   . 1

15 . 91

10 . 48

11 . 80

8   . 51

5  26

2   . 16

25 . 913

i) 13

5   . 24

7
a .26

4   .  19
1  .   14

0
0
0
5

5   . 24

1      2

1   . 83
5   . 24

3

1

5

56   . 315

0
83   . 522

0

6   . 40

0

3     20

0
0

22   . 78

0

9   .  29

140
141
142
143
144
145
146
147
148
150
151
152
153
154

155A
155B
156
157
158
159
160
161
162
164
170
171
172
173
174
175
176

692

693

MALIGNANT NEOPLASMS IN JAMAICA

TABLE VI-contd.

Site
177     Prostate
178   . Testis
179   . Penis

180   . Kidney   .

181   . Bladder, urethra, etc.

190   . Malignant melanoma of skini
191   . Skin
192   . Eye

193   . Nervous system
194     Thyroid gland

195   . Other endocrine glands
196   . Bone

197   . Connective tissue, muscle
198   . 2)in lymph node

199   . Other unspecified sites
200.0 . Reticulum cell sarcoma
200.1 . Lymphosarcoma

200.2 . Malignant lymphoma

unspecified

201   . Hodgkin's disease

202   . (iant follicular lymphoma
203   . Multiple myeloma

204.0 . C'hr. lymphatic leukaemia
204.1 . Chr. myeloid leukaemia
204.3 . Acute leukaemia

210   . Mixed salivary gland

140- . All sites
204

1958    1959   1960    1961   1962    1963   Total

M.

F.

M..

J .

M..
F.

M..

F .

M.

F..

M.
F.
M.
F.
M.
F.
M.
F.

M..

F.

M1.

F.

M..

F .

M.
F.
M.
F.
M.
F.
M.
F.

M..
F.

M..
F.

M..

F.

M..
F.

M..

F .

M..
F.

M..

F .

M..

F'.

M..
F.

M..

F .

10

1
11

2
3
5
5

2
14
16

2

2
1
1

1
5
4

3
5

1
3
1
2
1
1
2
1
1

5
1

15

2

7.
2.
4.
5.
5.
2.

2.

21

I

18

1.

2.
1 .

4.

3.
3.
1 .
3.
1 .
1 .
10
11

1.
5.

2.
1 .
3.
7.

1 .
1 .
6.
5.
1.
4.

9   .   11   .  11   .   16   .  72

0
2 .          .       .    -   .   5

0
10   .   10   .  10   .   14   .  62

0
3   .        .   8   .    3   .  18
2   .    1   .   2   .    1   .  13
2   .  12    .   5   .    7   .  36
5   .    3   .   4   .   10   .  32
1.          .    2.       2.      7
2.       4.      2.           .12
16   .  23    .  14   .   15   . 103
19   .   12   .  22   .  22    . 109

3.           .       .    2.      8
5   .    1   .   1   .    2   .  10
4   .    4   .   4   .    6   .  22
1   .   2   .    2   .   4   .   11

1I.     2   .    2.      8
8   .    2   .   2   .    4   .  20
- .  1 .     -   .    1  .    2

2   .        .   2
1   .   4   .    2   .   2   .   12

3.           .   2.       9
2   .    3   .   1   .    3   .  15
3   .    3   .   5   .    6   .  24
3.       3.          .    2.      9
4   .    2   .   1   .    2   .  11
11   .    9   .   3   .    5   .  41
19   .   15   .   8   .    8   .  66
1  .     1.     --   .    1  .    3
1  .     1  .   -    .    1  .    6
5   .    4   .   3   .    3   .  21

;>  I   .    6   .   4       1.]8
2   .   2   .    6   .   4   .   18

2                 ~~~~~~3

4

6   .    6   .   3   .    3   .27

1.     1- .        .        .   3
1 .              1      ..   . 3

2   .    2   .   1   .    3   .  10
3.          .    3.          .    9
9   .    )       1        1       6
2   .    3           .    1   .   9
1   .    2   .   2   .    3   .  10
1.      3.       1.       1.      6
2   .    5   .   1   .    3   .  22
5   .    2   .   2   .    4   .  19
3   .    4   .   1   .    1   .  10
4.       4.          .        .14
.  ---           -    .1100

-    .1798

I
I

I
I

694             G. BRAS, D. C. WATLER AND A. ASHMEADE-DYER

TABLE VII.-Compartson of Cancer Incidence in All Sites by Age Groups

Males                                   Females

.-A                          -r

K. & St. A.   Denmark     S.A. Bantu     K. & St. A.   Denmark     S.A. Bantu
(1958-1963)  (1943-1947)  (1953-1955)    (1958-1963)  (1943-1947)  (1953-1955)
Age groups     No. observed No. expected No. expected   No. observed No. expected No. expected

0-14     .      44                      44 0      .      31                        31-8

103-6                                     95

15-24     .      30J                      35-6     .      40J                      44-3
25-34     .      50           65-4        59*9     .     138          137-3        106 6
35-44     .      90          104-3        93-2     .     279          302-1        224-7
45-54     .     238          217-7       181-5     .     480          433-5        296-3
55-64     .     299         283-8        172-8     .     364          427-7        393.3
65-74     .     230         233 9        122-4     .     270          360-5        225-6
75+       .     104          188 7        63-8     .     165          367-7        112-1
Unknown .        15                                .      31

Total       .   1100         1197-4        773-2     .    1798        2123-8       1434-7

TABLE VIII.-Comparison of Cancer Incidence in Kingston and

St. Andrew (1958-1963) with Denmark (1943-1947)

Males                    Females

No. of cases Cases expected  No. of cases Cases expected
Site                K. & St. A. at Danish rates  K. & St. A. at Danish rates
Buccal cavity & pharynx  .   .   .     66          21-2     .     49          21-8
Oesophagus    .    .    .    .    .    87          29-9     .     39         24-2
Stomach .     .    .    .    .    .   160         236       .     91         268-7
Large intestinie ati(l rectur.  .  .   76         200       .    131         246-9
Liver (1?) .  .    .    .    .    .    26           3-6     .     11           3-3
Lung     .    .    .    .    .    .    83          80 3     .     24          24-3
Breast   .    .    .    .    .    .     5                   .    315         419-0
Cervix        .    .    .    .    .                         .    522         297-2
Body of uterus          .    .   .                          .     40          78-3
Testes   .    .    .    .    .    .     5          32-9     .      -           -
Penis    .    .    .    .    .    .    62           7-1     .

Skin     .    .    .    .    .    .   103          93.5     .    109          96-9
Leukaemias    .    .    .    .    .    38          56-4     .     34          53.4
Other sites   .    .    .    .   .    389         436.8     .    433        589.8

Total         .    .    .   1100       1197-7     .   1798        2123-8

primary site and by sex. Table VII compares the total cancer incidence with
those in Denmark and in the South African Bantu; Table VIII compares Jamaica
and Denmark regarding certain cancer types.

This Registry is supported by the British Empire Cancer Campaign for
Research.

REFERENCES

ROBERTS, G. W.-(1957) 'The Population of Jamaica'. Cambridge University Press.
CLEMMESEN, J. AND NIELSEN, A.-(1952) Acta. Un. int. Cancr., 8, 140.

HIGGINSON, J. AND OETTLE,, A. G.-(1960) J. natn. Cancer Inst., 24, 589.

				


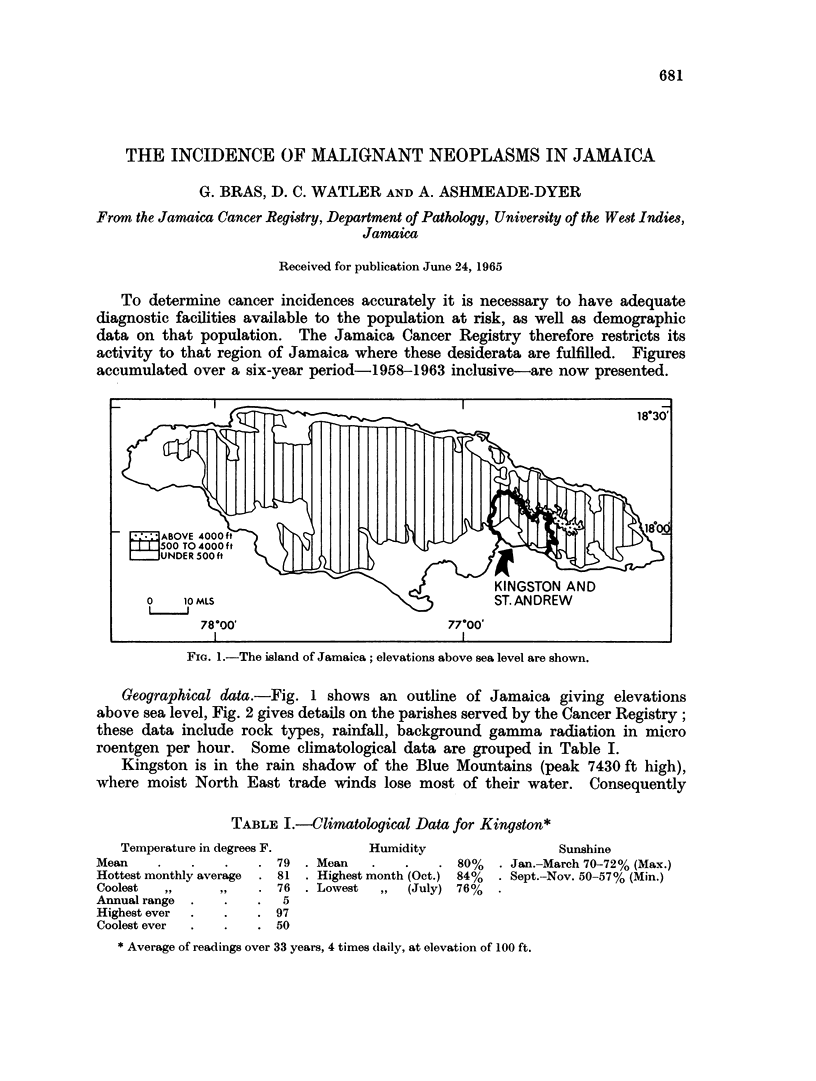

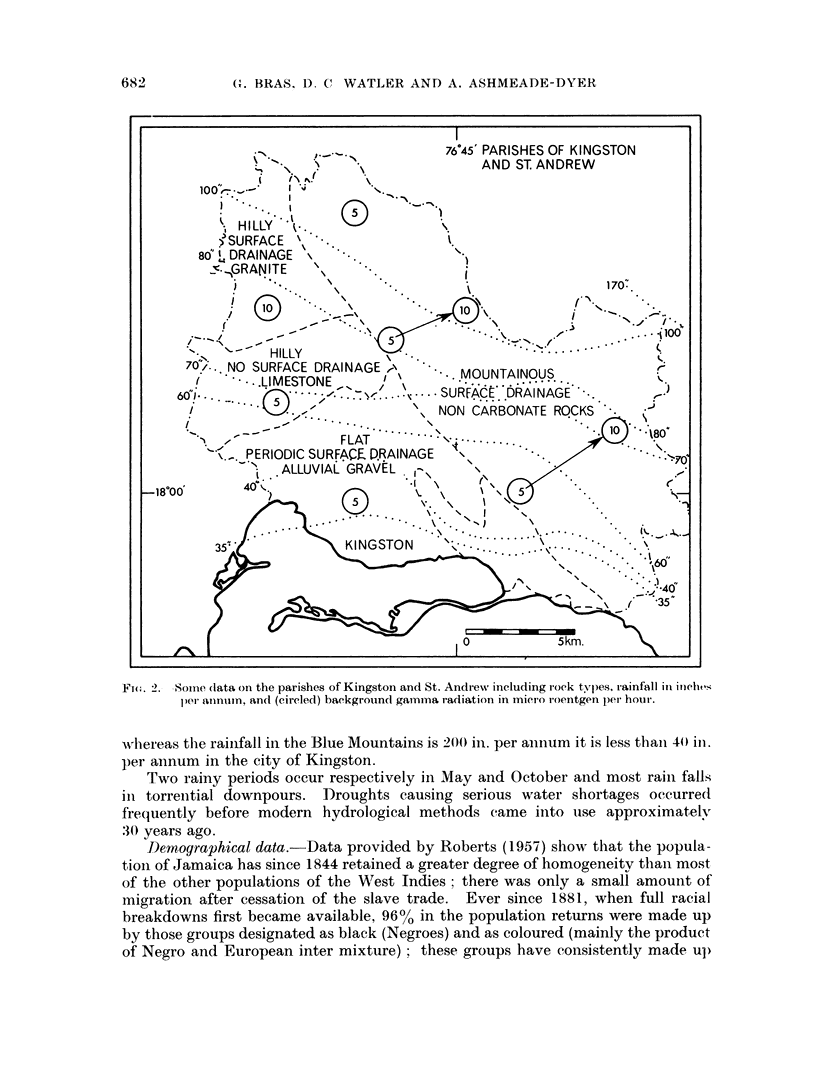

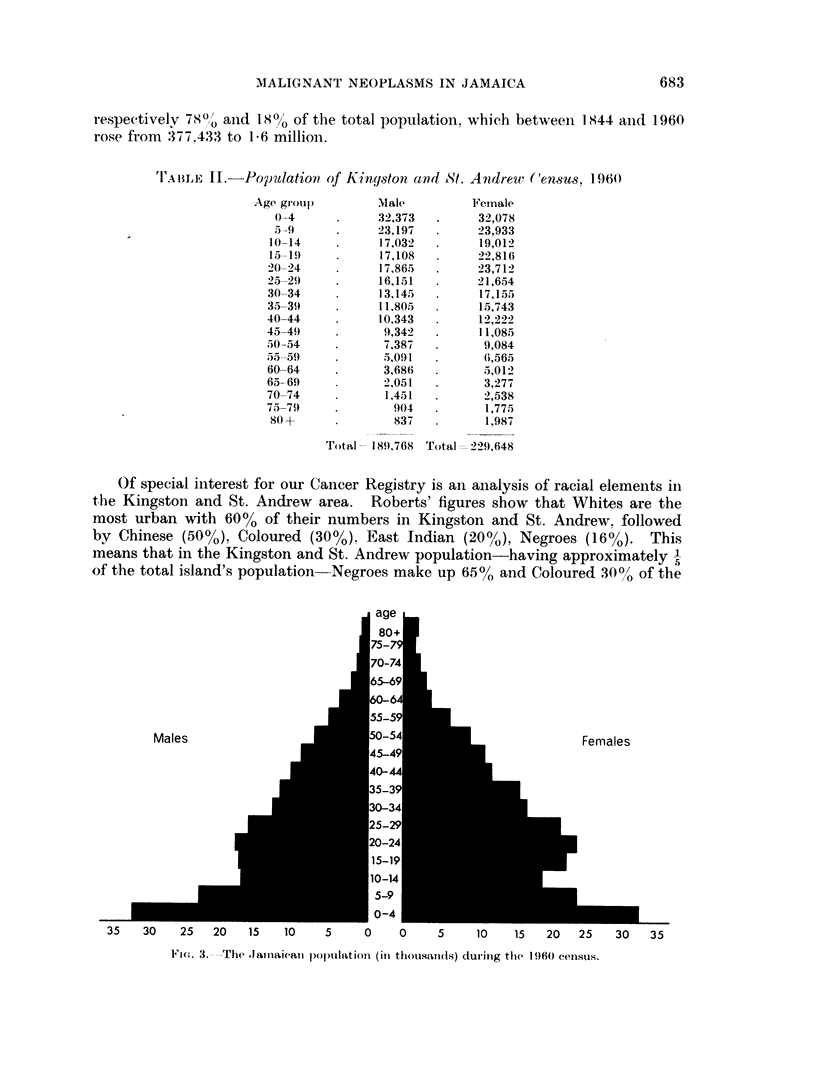

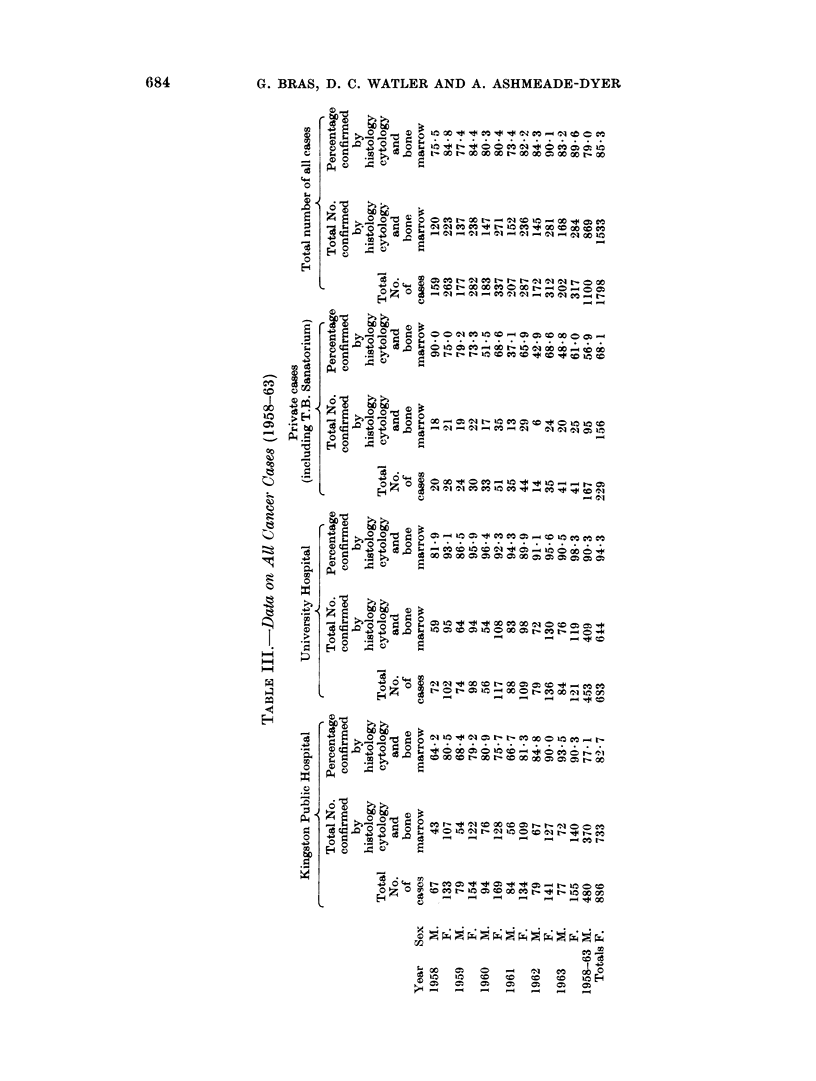

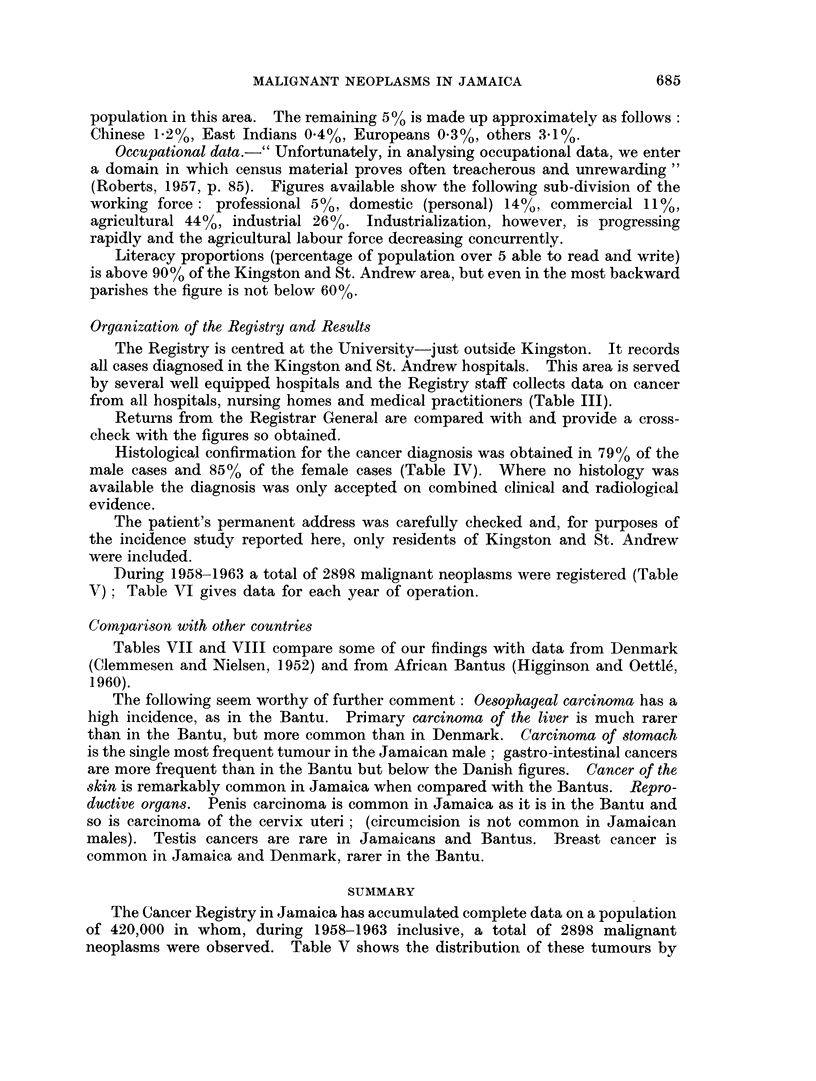

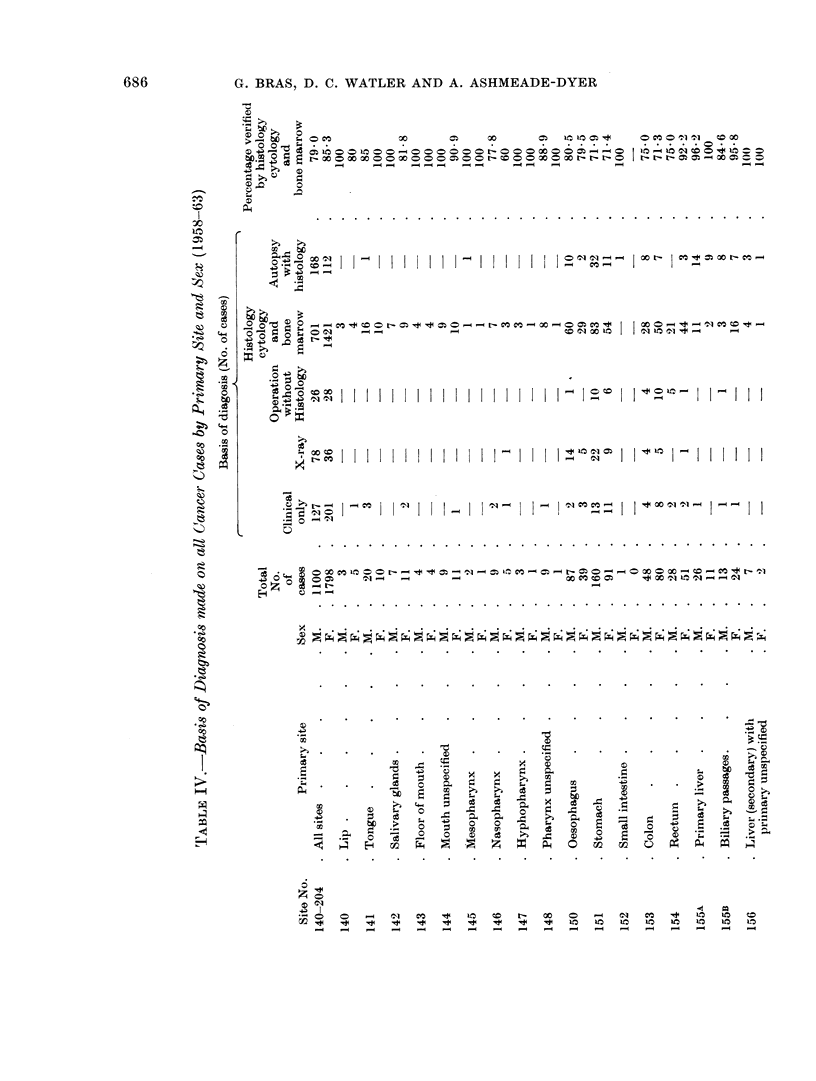

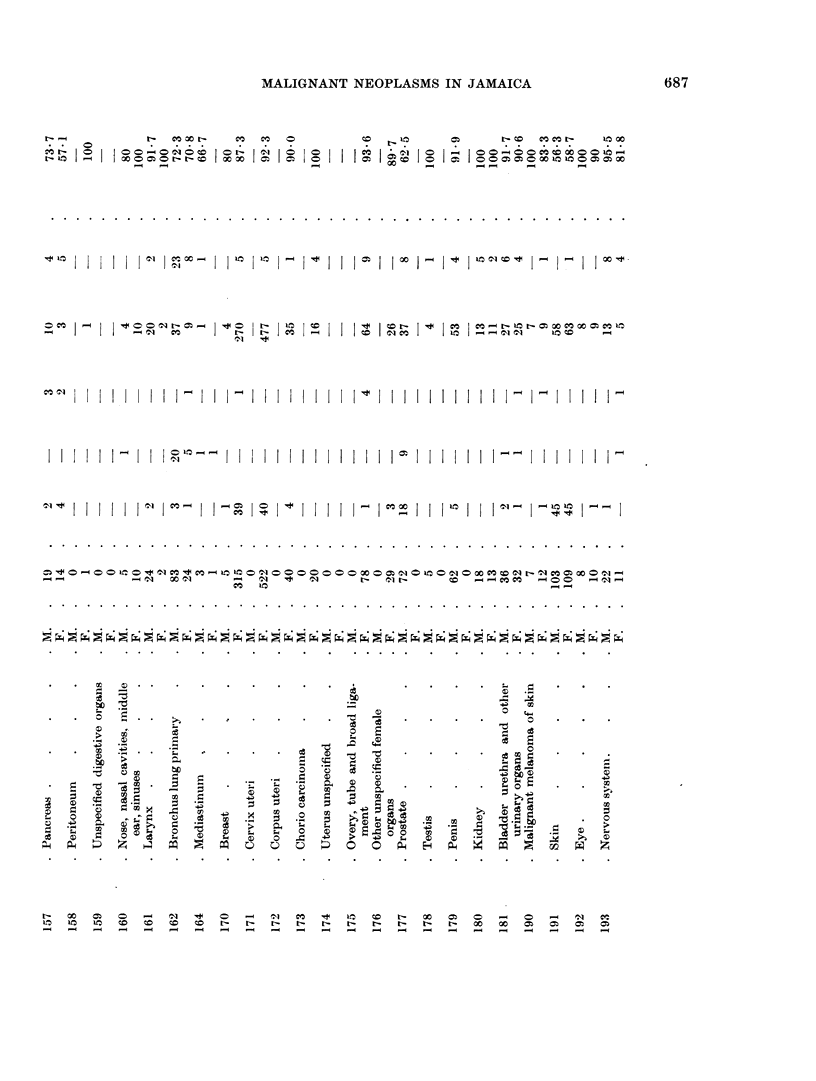

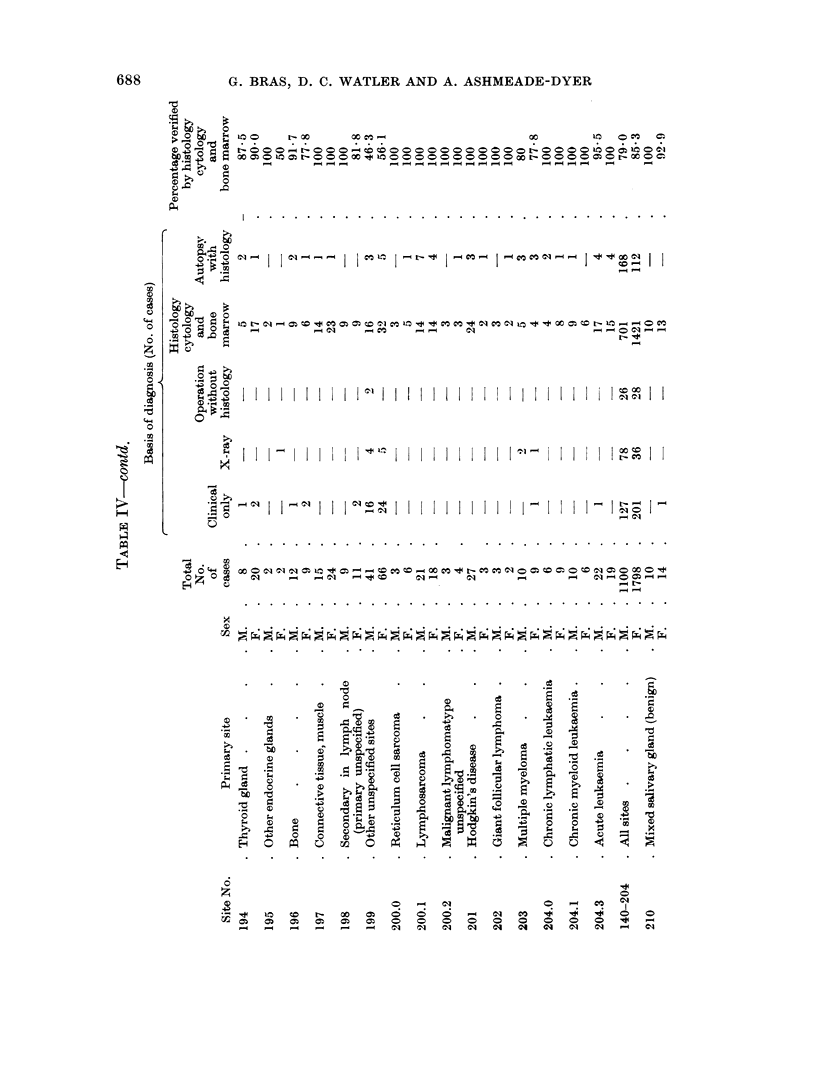

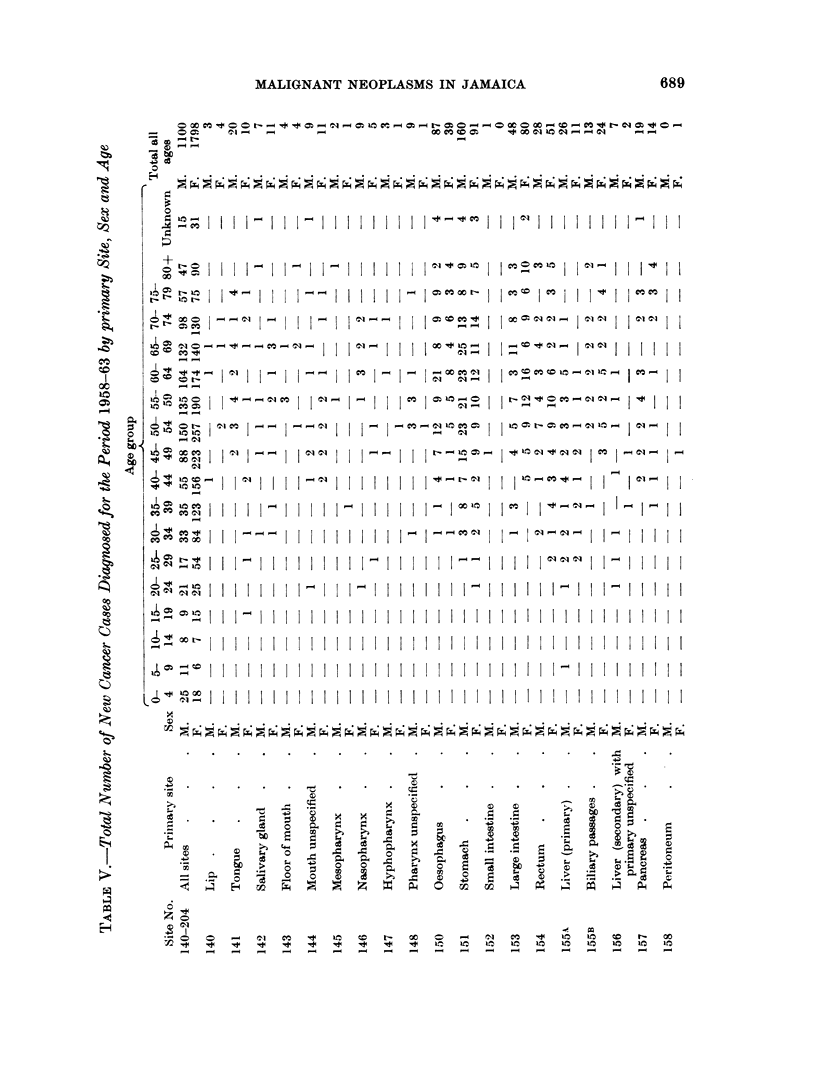

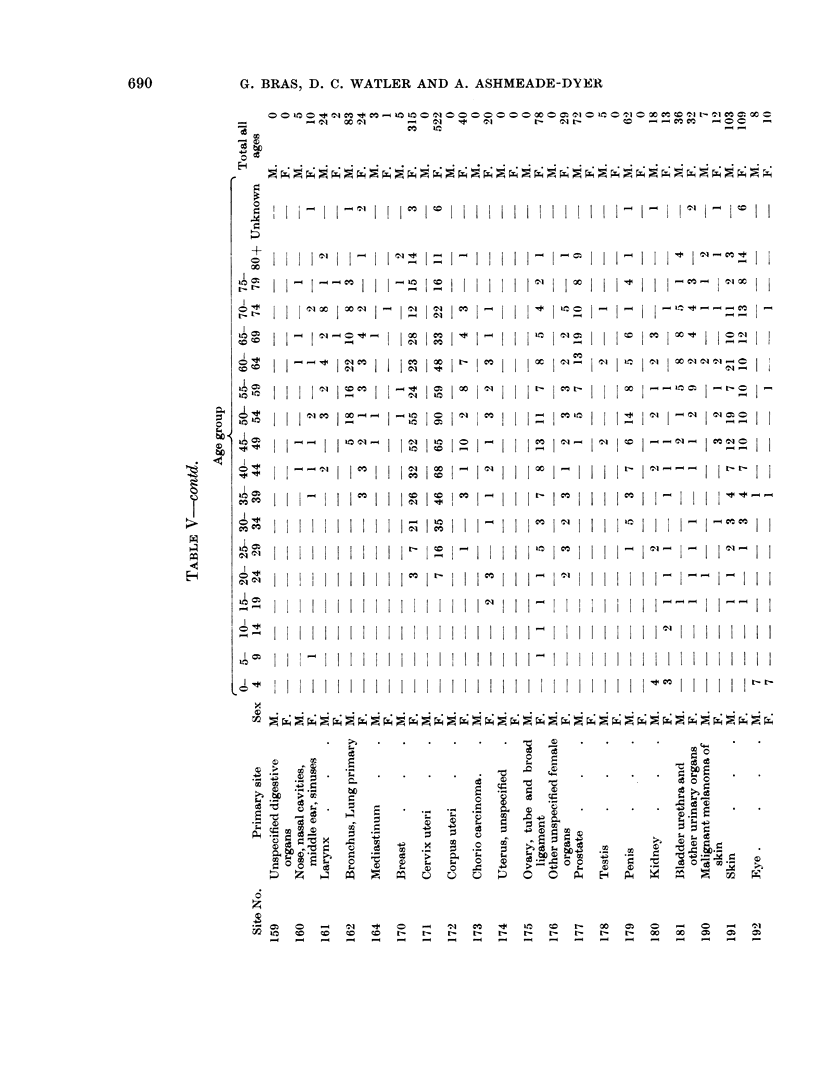

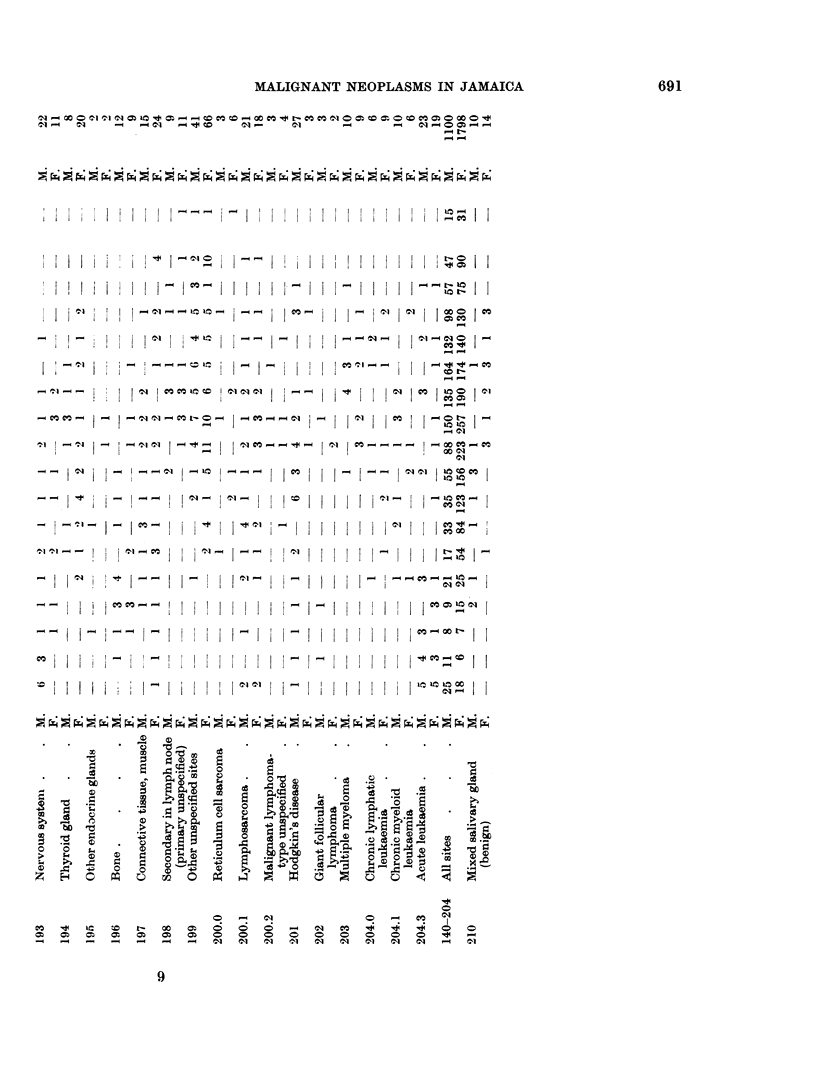

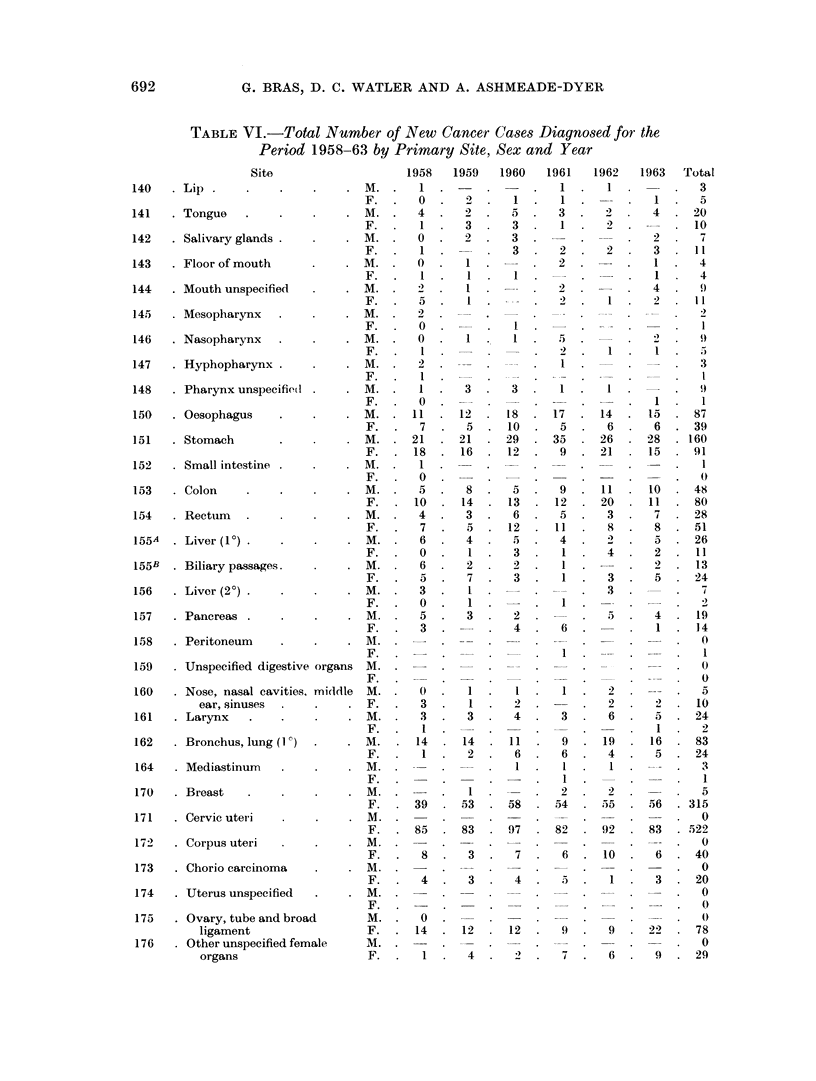

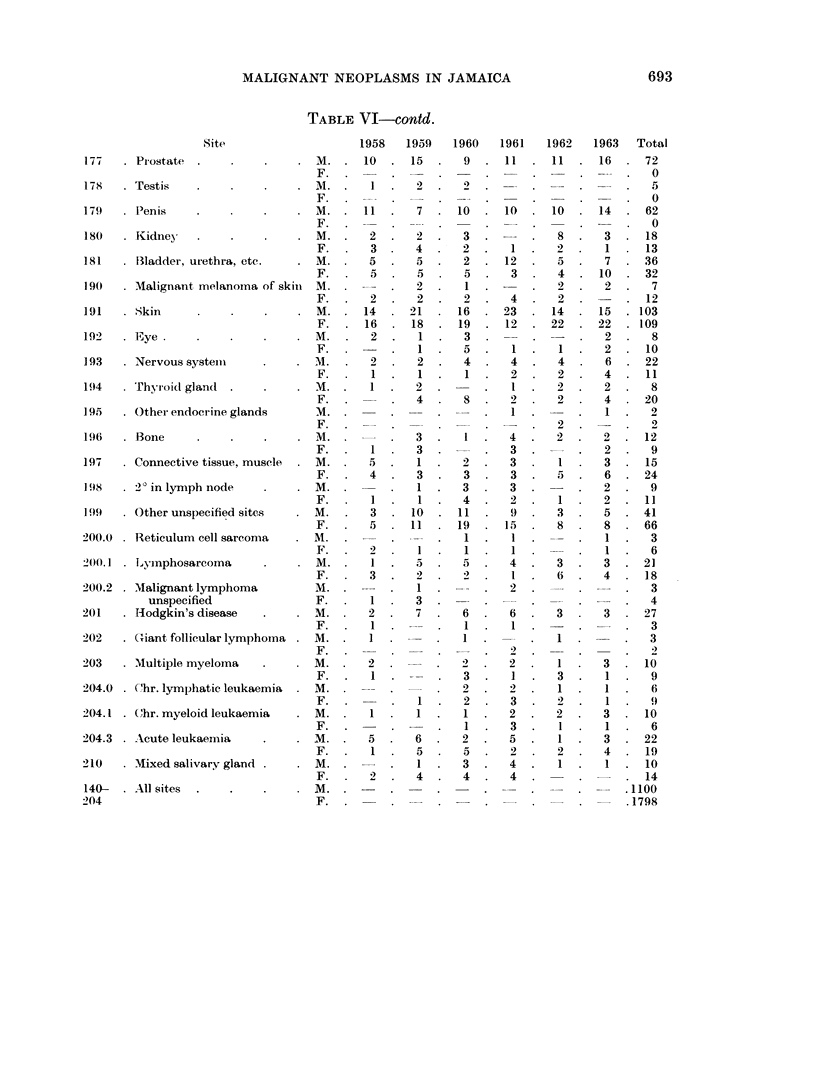

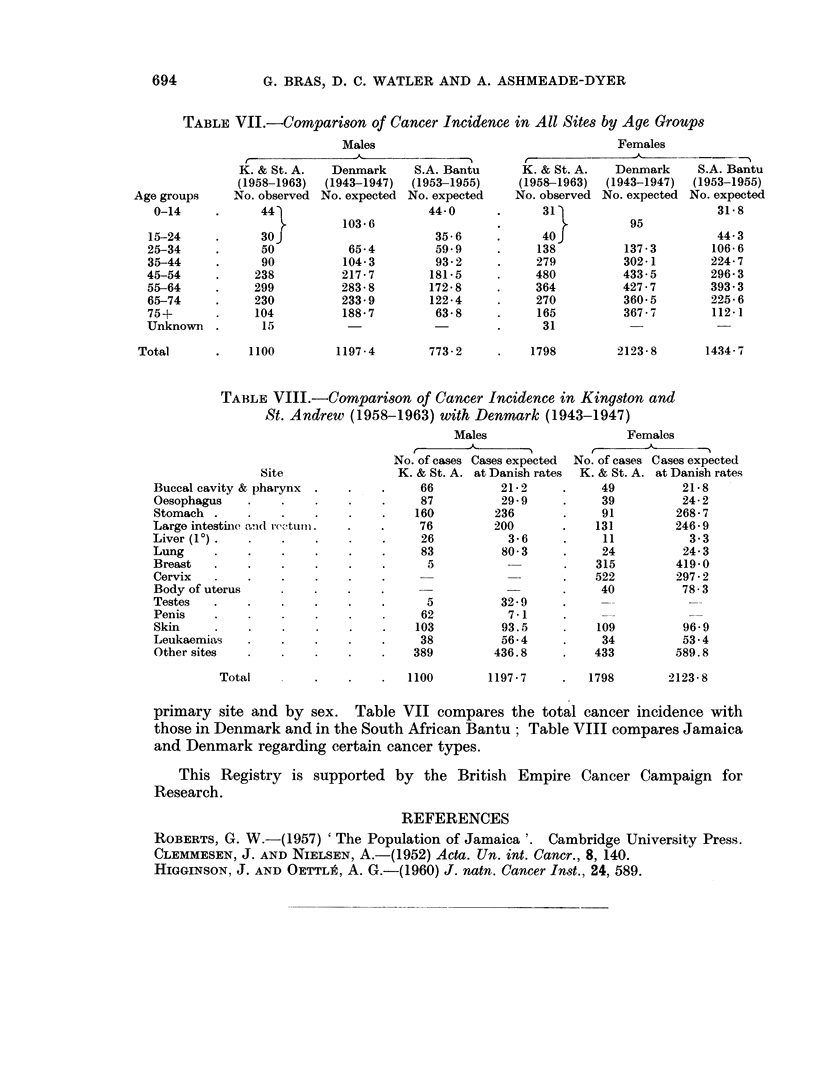

